# Lipopolysaccharide-induced innate immune responses are exacerbated by Prohibitin 1 deficiency and mitigated by S-adenosylmethionine in murine macrophages

**DOI:** 10.1371/journal.pone.0241224

**Published:** 2020-11-11

**Authors:** Soohan Jung, Jaehee Park, Kwang Suk Ko

**Affiliations:** 1 Department of Biomedical Sciences, College of Medicine, Seoul National University, Seoul, Korea; 2 Department of Nutritional Science and Food Management, College of Science and Industry Convergence, Ewha Womans University, Seoul, Korea; National Institutes of Health, UNITED STATES

## Abstract

Prohibitin 1 (Phb1) is a pleiotropic protein with multiple functions in mammalian cells including cell cycle regulation and mitochondrial protein stabilization. It has been proposed as a potential therapeutic target for a variety of diseases including inflammatory diseases. In this study, we investigated the potential immune-modulatory functions of Phb1 and anti-inflammatory properties of S-adenosylmethionine (SAMe) using macrophages, which play a major role in the innate immune system. The results showed that expressions of Phb1 mRNA and protein were reduced in lipopolysaccharide (LPS)-stimulated RAW 264.7 cells (*p*<0.05). Phb1 knockdown further ameliorated the mRNA expression of pro- and anti-inflammatory cytokines such as TNF-α, IL-1α, IL-1β, IL-6, and IL10 in LPS-stimulated RAW 264.7 cells. SAMe significantly attenuated LPS-induced inflammatory responses such as IL-1β, IL-10, Nos2, and NO production in the presence of *siPhb1*. Luciferase reporter assay was conducted to determine the mechanisms underlying the effects of Phb1 and SAMe on the immune system. The luciferase activity of nuclear factor kappa-light-chain-enhancer of activated B cells (NF-κB) was significantly increased in LPS-treated RAW 264.7 cells. In addition, the luciferase reporter assay showed increased NF-κB activation in Phb1 knockdown RAW 264.7 cells (*p*<0.1) and SAMe treatment attenuated the NF-κB luciferase activity in Phb1 knockdown RAW 264.7 cells. Based on the results, we concluded that Phb1 possibly modulates the inflammatory response whereas SAMe has an anti-inflammatory effect on Phb1 knockdown macrophage cells. Furthermore, Phb1 expression level has potential properties of affecting on innate immune system by modulating the NF-κB signaling pathway.

## Introduction

Prohibitin 1 (Phb1) is a ubiquitously expressed protein in eukaryotic cells and exhibits a high degree of sequence homology among species [1]. Phb1 functions as a chaperone protein that stabilizes the proteins created in mitochondria [2]. It is also involved in cell cycle control, differentiation, apoptosis, senescence, and cell fate determination [2]. Alteration of Phb1 levels has been associated with pathologies including inflammation, autoimmunity, and cancer [2]. The relationship between Phb1 and immune functions might be in association with IgM receptor, one of the two major classes of antigen receptors on murine B lymphocytes [3]. However, the function of Phb1 in IgM receptor immune signaling has barely been explored. More recently, Phb1 was identified as an important component of antigen-mediated signaling in mast cells, in the adaptor molecules in B cell receptors, and in the maturation of T cells [4–6]. The levels of Phb1 mRNA and protein were found to be lower in the inflamed mucosa of individuals with inflammatory bowel disease (IBD) as well as in experimental models of colitis [7, 8]. Tumor necrosis factor alpha (TNF-α), a key cytokine that plays a central role in the immune response, reduces Phb1 levels in cultured intestinal epithelial cells [9]. Collectively, these results suggest that Phb1 has a potential to modulate the inflammatory responses.

S-adenosylmethionine (SAMe) is an important molecule in all living cells. It is not only involved in the *de novo* synthesis of cellular glutathione (GSH), a strong endogenous antioxidant, which also serves as a fundamental biological methyl donor [10–12]. SAMe is also known to possess anti-inflammatory properties and exhibits a therapeutic effect in osteoarthritis [13]. Exogenous SAMe supplementation downregulates the expression of pro-inflammatory cytokines induced by lipopolysaccharide (LPS) stimulation such as TNF-α and IL-6 [14]. According to Moon et al, SAMe suppresses the IκB kinase (IKK) / nuclear factor kappa-light-chain-enhancer of activated B cells (NF-κB)-mediated inflammatory responses induced by TNF-α in adipocytes [15]. Therefore, the alteration of Phb1 levels is closely related to the inflammatory response and SAMe can be a useful supplementary treatment in inflammation. However, the roles of Phb1 and SAMe as well as their relationship in immune cells such as macrophages are not well known. Thus, the aim of this study is to investigate the potential roles of the immune modulatory function of Phb1 and to identify the anti-inflammatory properties of SAMe using murine macrophages.

## Material and methods

### Cell culture and treatments

The murine macrophage cell line, RAW 264.7, was purchased from ATCC (Manassas, VA, USA). The cells were cultured in Dulbecco’s Modified Eagle’s Media (DMEM; HyClone Laboratories INC., Logan, UT, USA) supplemented with 10% (v/v) fetal bovine serum (FBS; Gibco Inc., Grand Island, NY, USA) and 1.5 g/L sodium bicarbonate (Daejung Chemicals Co., Siheung, Korea) at 37°C in a humidified 5% CO_2_ incubator. Cells with passage numbers between 10 and 15 were used for the experiments and seeded into 6-well plates at a density of 0.2 x 10^6^ cells/well for 6 hours before treatments. After 6 hours, the media were changed with the new media containing 1 mM SAMe (Samoh Pharm Co., LTD, Seoul, Korea), followed by 24 hours of incubation. Afterward, the cells were stimulated with 1 μg/mL LPS (Sigma-Aldrich Co., St. Louis, MO, UA) for 4 hours.

### Small interfering RNA (*siRNA*) transfection

Pre-designed *siRNA* targeting mouse Phb1 (sense: AGAGCGAGCGGCAACAUUUTT, antisense: AAAUGUUGCCGCUCGCUCUGT) and nonspecific scrambled *siRNA* were purchased from Ambion Inc. (Austin, TX, USA). RAW 264.7 cells were plated on 6-well plates and transfected with 13 nM *siPhb1* or scramble *siRNA* using Lipofectamine RNAiMAX (Invitrogen Inc., Carlsbad, CA, USA) according to the manufacturer’s manual.

### Cell viability

The tetrazolium-based colorimetric assay (MTT assay) was used to measure cell viability. At the end of treatments, 3-[4,5-dimethylthiazole-2-yl]-2,5-diphenyltetrazolium bromide (MTT) solution (Sigma-Aldrich, St. Louis, MO, USA) was added to each well with the cells and incubated for 4 hours. Then the supernatant was removed and 100 μL of dimethyl sulfoxide (DMSO, Amresco Co Ltd., CITY, Ohio, USA) was added to dissolve the formazan. The absorbance was read at 570 nm using a microplate reader (EZ Read 400, Biochrom LTD, Cambridge, UK).

### RNA isolation and quantitative real time PCR

Total RNA isolation was performed using TRIzol reagent (Invitrogen, Carlsbad, CA, USA) following the manufacturer’s instructions. For cDNA synthesis, First Strand cDNA Synthesis Kit (Thermo Fisher Scientific, Waltham, MA, USA) was used following the manufacturer’s guide. Quantitative real time PCR (qPCR) was performed with the Maxima SYBR Green qPCR Master Mix (Thermo Fisher Scientific, Waltham, MA, USA) and PikoReal 96 Real Time PCR System (Thermo Fisher Scientific). The primers used for this experiment are shown in Table 1 and all qPCR reactions were performed in duplicate for each sample. The relative gene expression levels were analyzed using the Ct method and mouse beta actin was used as an internal control for normalization of the relative gene expression.

**Table 1:** List of primer sequences for quantitative real time PCR.

Gene name	Reference sequence	Primer sequence	
Actin, beta	NM_007393.5	Forward	GGTATCCTGACCCTGAAGTA
		Reverse	CACACGCAGCTCATTGTA
Phb1	NM_008831.4	Forward	GTGGTGAACTCTGCTTTGTA
		Reverse	CCAAGGGATGAGGAAATGAG
TNF-α	NM_001278601	Forward	CCTATGTCTCAGCCTCTTCT
		Reverse	GGGAACTTCTCATCCCTTTG
IL-1α	NM_010554.4	Forward	CCTGTAACAGACCTCAAGAAGG
		Reverse	CCGTCAAGCTCAGAGGATTT
IL-1β	NM_008361.4	Forward	TCACAAGCAGAGCACAAG
		Reverse	GAAACAGTCCAGCCCATAC
IL-6	NM_001314054.1	Forward	CTTCCATCCAGTTGCCTTCT
		Reverse	CTCCGACTTGTGAAGTGGTATAG
Nos2	NM_001313921.1	Forward	GTCTGCATGGACCAGTATAAG
		Reverse	GGTGTGGTTGAGTTCTCTAAG
IL-10	NM_010548.2	Forward	TGAATTCCCTGGGTGAGA
		Reverse	CCACTGCCTTGCTCTTATT

### Nitric oxide assay

Nitric oxide (NO) concentration in the cells was measured using the Griess method. Cells transfected with *siPhb1* or scrambled *siRNA* were seeded on 6-well plates at a density of 0.2 x 10^6^ cells/well. After 24 hours, the cells were stimulated with LPS for 4 hours followed by an NO assay. The media were transferred to a 96-well plate and an equal volume of prepared Griess reagent was added to each well. After 10 minutes of incubation, the absorbance was measured at 562 nm.

### Western blot analysis

Total protein was extracted using radioimmunoprecipitation (RIPA) buffer containing 150 mM NaCl, 1% NP-40, 1 mM EDRA, 0.25% deoxycholic acid, and 50 mM Tris (pH 7.4). The amount of protein (20 μg/well) was separated by 12.5% SDS-polyacrylamide gel electrophoresis and transferred to a nitrocellulose membrane, followed by Ponceau S staining to confirm equal loading of the protein. After blocking with 5% skim milk in 0.1% TBS-Tween 20 for 1 hour at room temperature, the membranes were incubated with the primary Phb1 antibody (Cell Signaling Technology, Beverly, MA, USA) for 12 hours at 4°C. The membranes were further incubated with horseradish peroxidase-conjugated anti-rabbit immunoglobulin G secondary antibodies (Cell Signaling Technology, Beverly, MA, USA) for 1 hour at room temperature. The immunoreactive bands were visualized using enhanced chemiluminescence (ECL) solution (LPS Solution, Daejeon, Korea) on a Kodak X-OMAT 2000 X-ray film processor (Kodak, Rochester, NY, USA) and alpha-tubulin was used as an internal control to quantify the relative protein expression.

### Reporter assay

To determine the promoter activation of NF-κB with a reporter assay, RAW 264.7 cells were seeded in 6-well plates at a density of 0.2 x 10^6^ cells/well for 6 hours before treatments. After 6 hours, the media were changed to media containing 1 mM SAMe for 24 hours then stimulated for 4 hours with 1 μg/mL LPS. At the end of the treatment, the cells were washed three times with PBS and processed according to the manufacturer’s instructions. The Dual-Glo Luciferase Reporter Assay System (Promega, Madison, WI, USA) was used to quantify the expression of the firefly luciferase and the Renilla luciferase. The firefly luciferase was normalized to the Renilla and presented relative to the controls.

### Statistical analysis

All data are expressed as mean standard errors (SEM) and were analyzed using the Statistical Analysis System package version 9.3 (SAS Institute, Cary, NY, USA). Differences among groups were determined by one-way analysis of variance (ANOVA) with Duncan’s test. Values of *p* < 0.05 were considered statistically significant.

## Results

### Effect of LPS stimulation on Phb1 expression in RAW 264.7 cells

**Fig 1:**
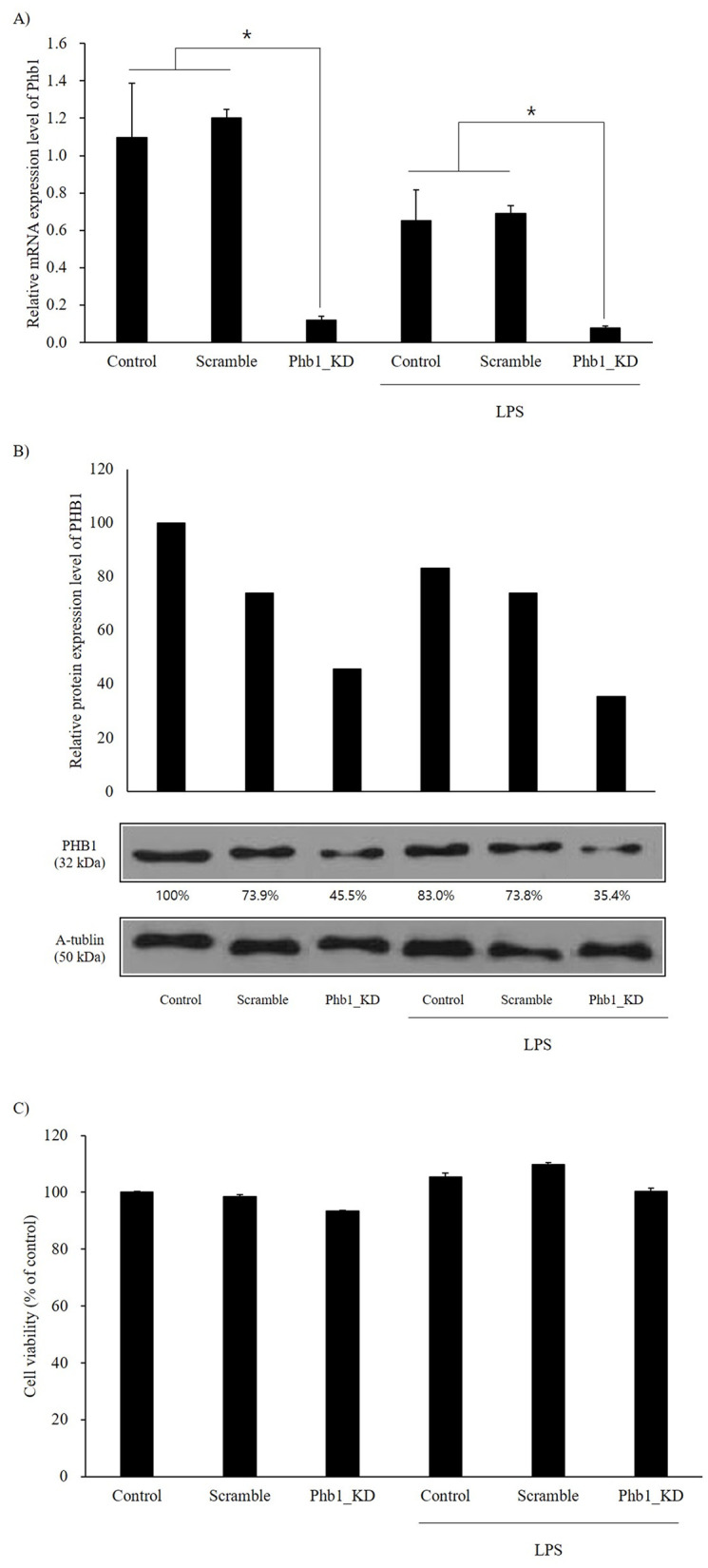
A: Relative mRNA expression level of RAW 264.7 cells stimulated with LPS after siPhb1 RNA transfection. B: Protein expression of PHB1 by western blots. C: Cell viability of each group of RAW 264.7 cells. Values represent the means ± SE of three replicate experiments performed with independent cultures. * Indicate significant differences by one-way ANOVA (*p*<0.05).

To determine the effect of LPS stimulation on Phb1 expression in macrophages, we measured relative mRNA expression level and protein level of Phb1. The relative mRNA expression level of *Phb1* was significantly decreased in LPS-induced RAW 264.7 cells compared to the level in normal cells (Fig 1A). Western blot analysis also showed a comparable reduction in the PHB1 protein level in LPS-induced cells (Fig 1B). The knockdown efficiency of *siPhb1* in this experiment was greater than 80% (Fig 1A and 1B).

### Cell viability & proliferation

As shown in Fig 1C, the cell viability was slightly reduced in Phb1- knockdown RAW 264.7 cells (*p*<0.05). Four hours of LPS stimulation resulted in a significant increase of RAW 264.7 cell proliferation (*p*<0.05).

### Cytokine expression

**Fig 2:**
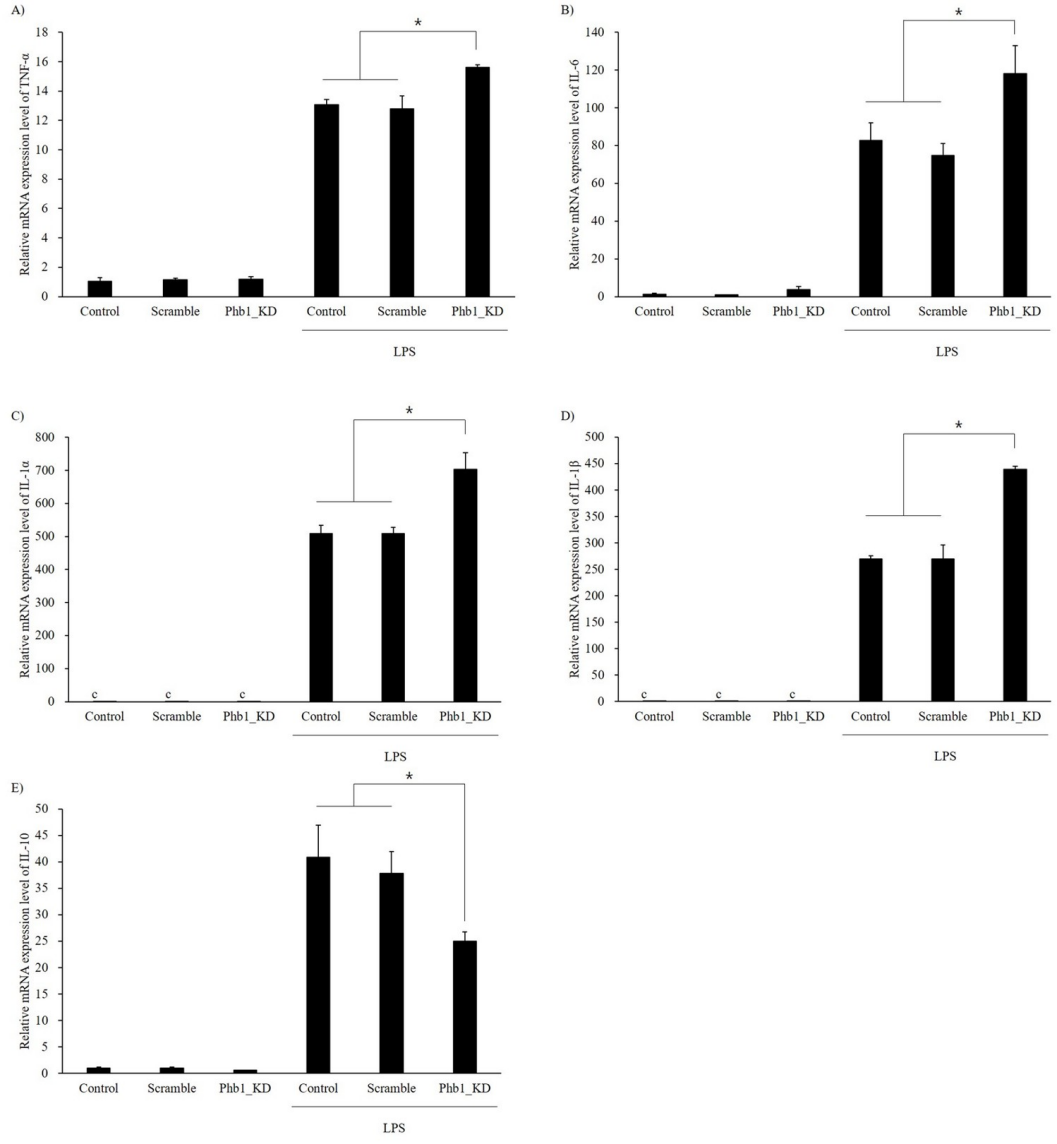
Effects of Phb1 expression level and LPS stimulation on inflammatory cytokines in RAW 264.7 cells with Phb1 knockdown. A: TNF-α, B: IL-6, C: IL-1α, D: IL-1β, E: IL-10. Values represent means ± SE of three replicate experiments performed with independent cultures. * Indicate significant differences by one-way ANOVA (*p*<0.05).

The relative mRNA expression levels of the pro-inflammatory cytokine genes after LPS stimulation are shown in Fig 2. Pro-inflammatory cytokines such as TNF-α, IL-6, IL-1α, and IL-1β were highly expressed in LPS-stimulated groups compared with those of the non-stimulated groups. Interestingly, the mRNA expression levels of pro-inflammatory cytokines were more elevated in the *siPhb1*-transfected cells than in the normal cells (*p*<0.05). The mRNA expression level of the anti-inflammatory cytokine IL-10 also increased in the LPS-stimulated groups compared with those of the non-stimulated groups (Fig 2E). However, when the *Phb1* mRNA expression level was downregulated by *siPhb1*, the mRNA expression level of IL-10 after LPS stimulation was significantly decreased compared with cells that expressed normal levels of Phb1 (Fig 4E, *p*<0.05).

### Nitric oxide production and Nos2 expression

**Fig 3:**
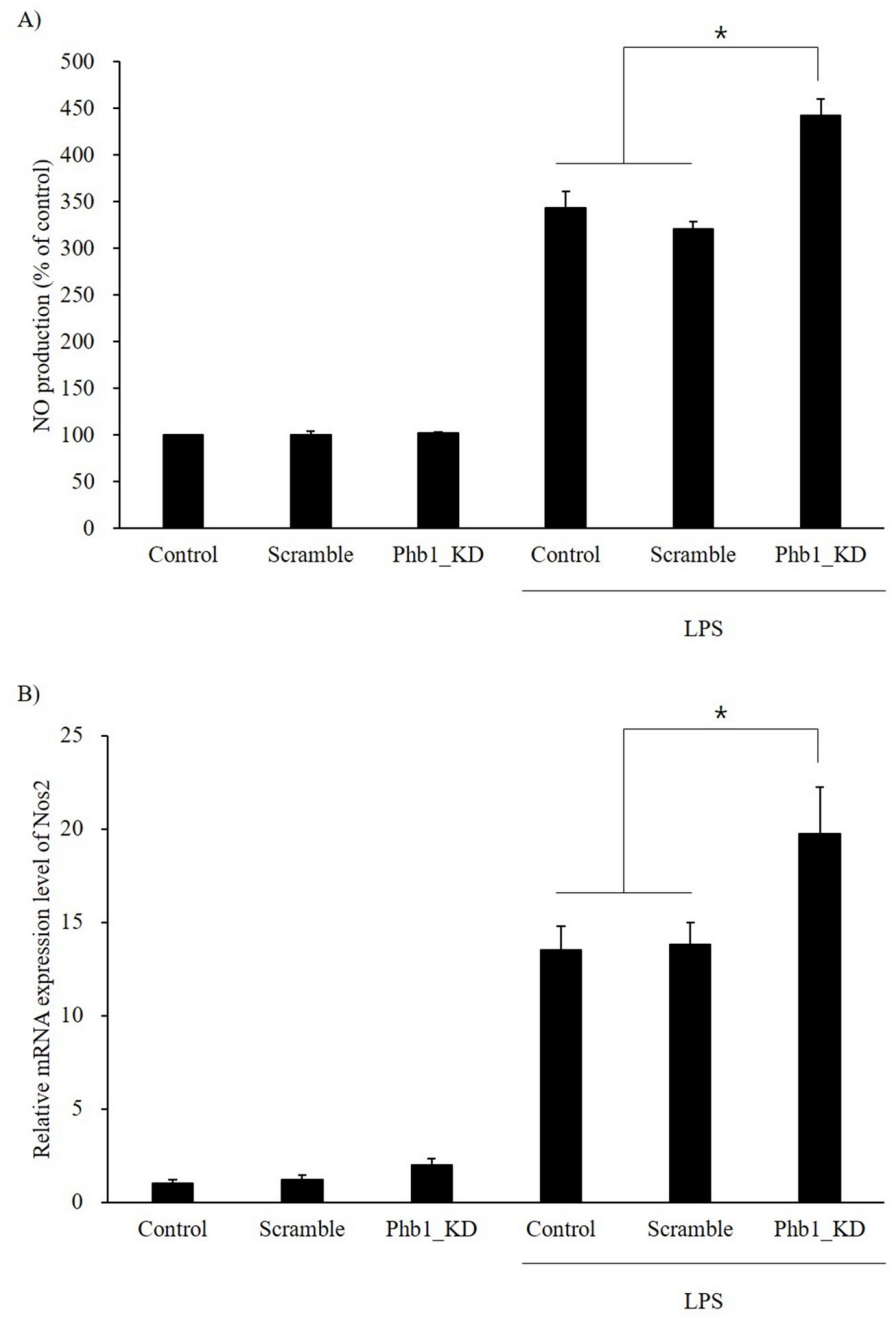
Alteration of A: NO production and B: mRNA expression level of Nos2 in LPS-stimulated RAW 264.7 cells with Phb1 knockdown. Values represent means ± SE of three replicate experiments performed with independent cultures. * Indicate significant differences by one-way ANOVA (*p*<0.05).

LPS stimulation increased NO production in RAW 264.7 cells in both normal and Phb1-knockdown cells. Furthermore, NO production and the relative mRNA expression levels of *Nos2* were significantly increased in *siPhb1*-transfected RAW 264.7 cells compared with those of the other groups (Fig 3).

### Effects of SAMe on Phb1 expression in LPS-stimulated RAW 264.7 cells

**Fig 4:**
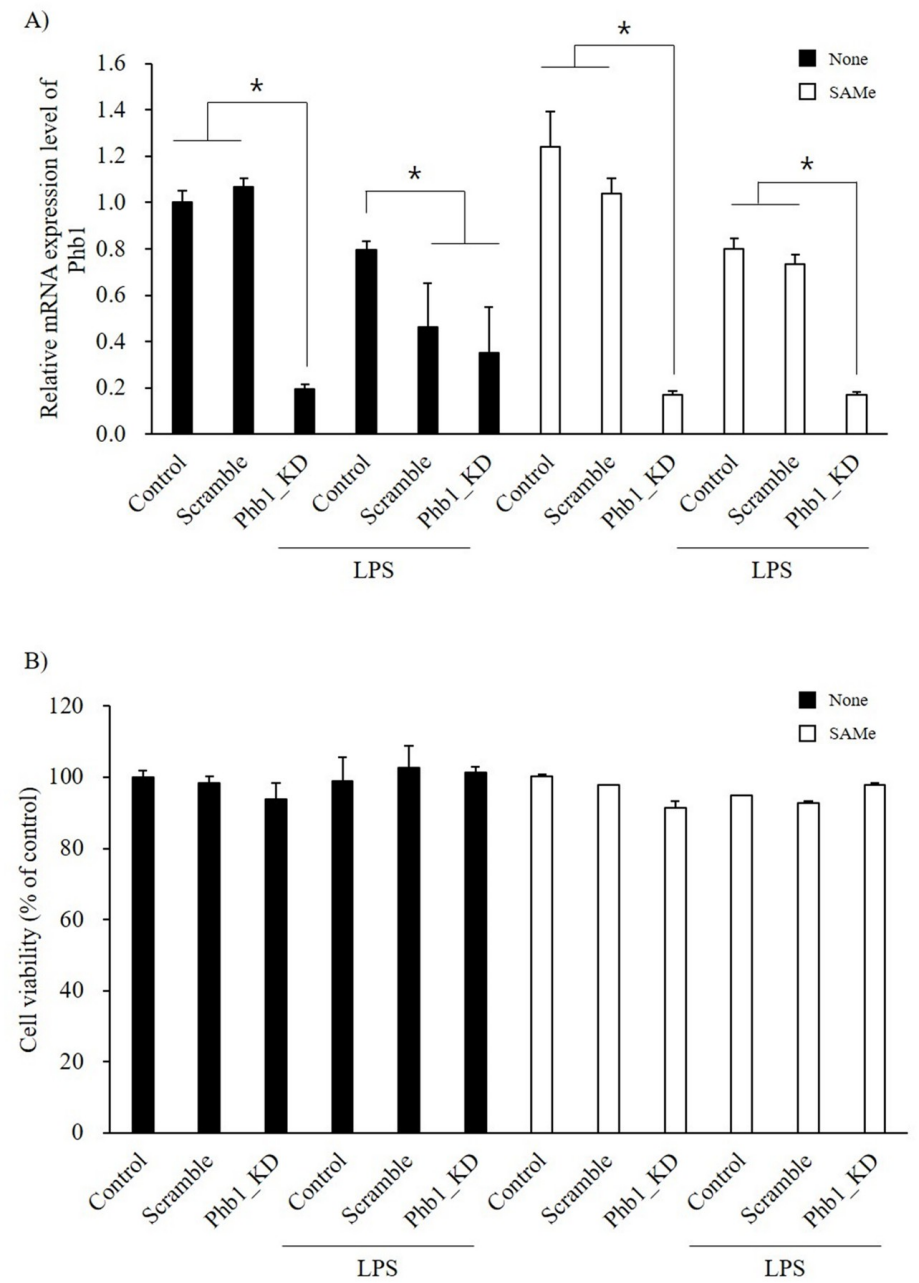
Relative mRNA expression level of Phb1 in RAW 264.7 cells (A) treated with SAMe before LPS stimulation and its cell viability (B). Values represent means ± SE of three replicate experiments performed with independent cultures. * Indicate significant differences by one-way ANOVA (*p*<0.05).

After confirming that LPS induced Phb1 downregulation as shown in Fig 1, we further attempted to determine the effect of SAMe on Phb1 expression in LPS-stimulated RAW 264.7 cells. The Phb1 expression in normal RAW 264.7 cells tended to increase in SAMe-treated cells (Fig 4). However, the results showed that the *Phb1* mRNA expression was not altered by SAMe treatment in the LPS treatment groups (Fig 4A).

### SAMe treatment and cell viability

The effects of SAMe on cell viability were shown in Fig 4B. The cell viability of RAW 264.7 cells without SAMe was similar to that shown in Fig 1. The SAMe-treated groups showed no significant differences in cell viability except for the SAMe-treated Phb1-knockdown group. This group showed significantly reduced cell viability compared with the other groups (*p*<0.05).

### SAMe treatment and cytokine expression

**Fig 5:**
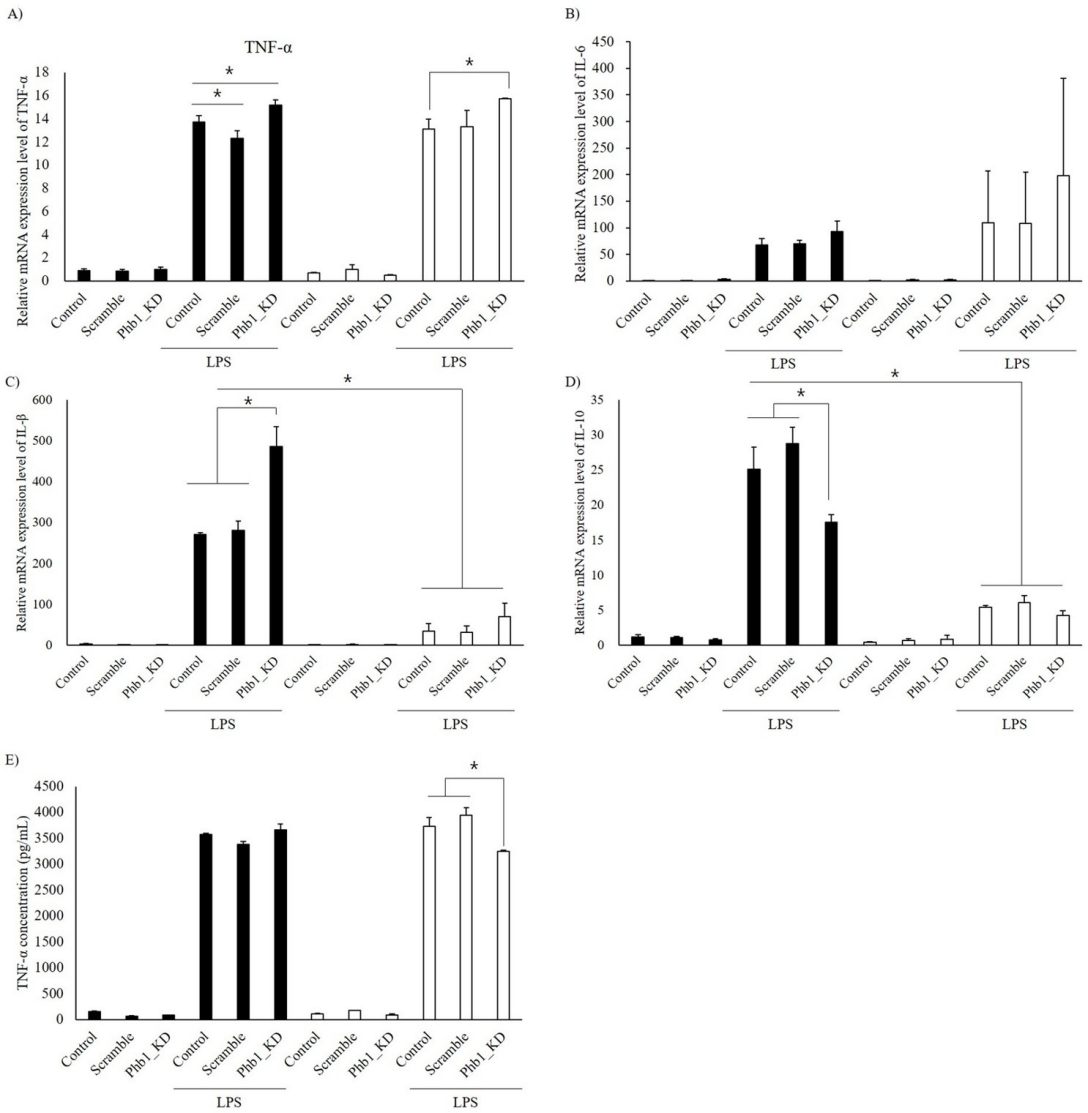
Effect of SAMe on mRNA expression levels of inflammatory cytokines in RAW 264.7 cells with Phb1 knockdown and LPS stimulation. A: TNF-α, B: IL-6, C: IL-1β, D: IL-10. Values represent means ± SE of three replicate experiments performed with independent cultures. * Indicate significant differences by one-way ANOVA (*p*<0.05).

The mRNA expression levels of inflammatory cytokines were determined in RAW 264.7 cells with or without SAMe. The mRNA expression levels of TNF-α and IL-6 showed no statistical differences with and without SAMe treatment. However, the mRNA expression levels of IL-1β and IL-10 were decreased significantly in the SAMe-treated LPS-induced RAW 264.7 cells compared with the LPS-induced only cells (Fig 5C and 5D). The extracellular secreted level of TNF-α was determined by ELISA assay (Fig 5E). The result of ELISA was similar with relative mRNA expression level of TNF-α in Fig 5A. However, in SAMe treated Phb1 KD group with LPS stimulation showed significant decreased TNF-α concentration compared with other SAMe treated LPS stimulation groups (*p*<0.05).

### Effects of SAMe treatment on nitric oxide production and Nos2 expression

**Fig 6:**
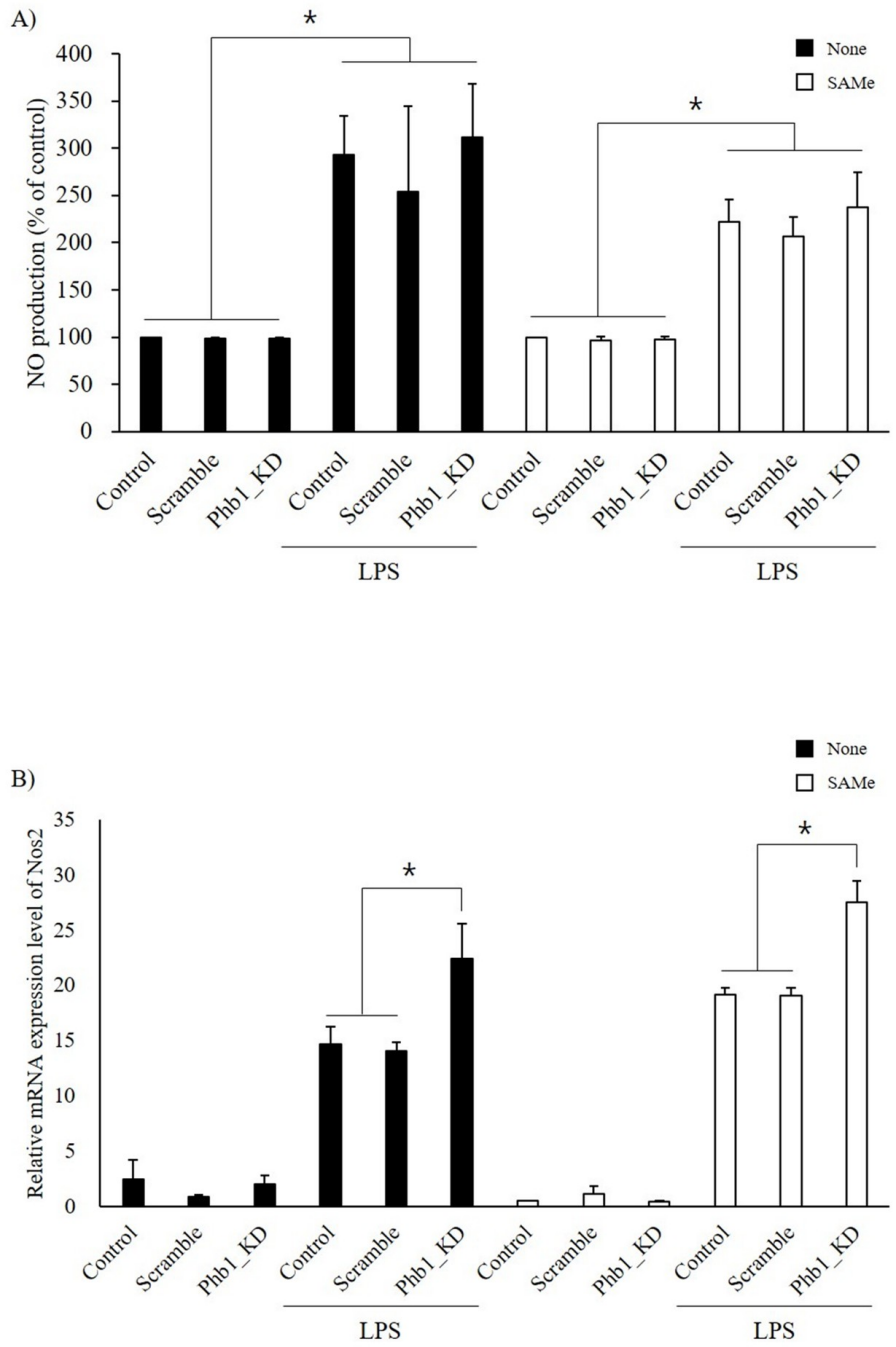
The NO production (A) and mRNA expression level of Nos2 (B) in SAMe-treated RAW 264.7 cells. After SAMe treatment, cells were transfected with or without siPhb1 RNA then stimulated with LPS. Values represent means ± SE of three replicate experiments performed with independent cultures. * Indicate significant differences by one-way ANOVA (*p*<0.05).

The effects of SAMe on NO production and *Nos2* expression level in LPS-stimulated RAW 264.7 cells were shown in Fig 6. Pretreatment with SAMe significantly attenuated the LPS-induced NO production and *Nos2* expression (*p*<0.05). Especially, the reduction of the LPS-induced *Nos2* expression by SAMe treatment was lower in the *siPhb1* and LPS group than in the control group (Fig 6B).

### NF-κB activation

**Fig 7:**
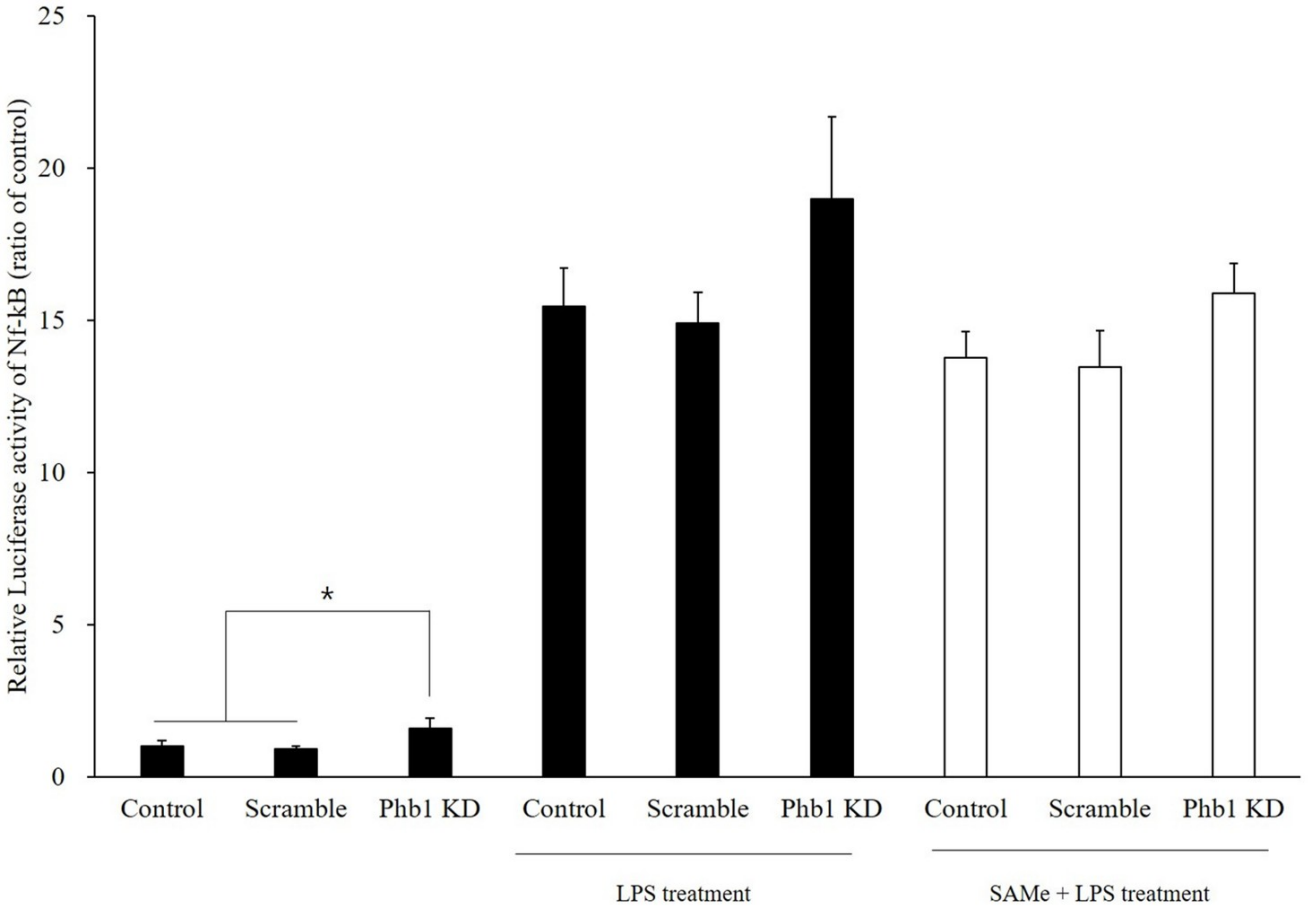
Modulation of LPS-induced NF-κB activation by SAMe in Phb1-knockdown RAW 264.7 cells. Values represent means ± SE of three replicate experiments performed with independent cultures. * Indicate significant differences by one-way ANOVA (*p*<0.05).

To determine the effects of Phb1 and SAMe on NF-κB activation, luciferase reporter assay was conducted. In Fig 7, luciferase activity of NF-κB was observed in the cells stimulated with LPS, and it was clear that LPS treatment activated the RAW 264.7 cells. In addition, the luciferase reporter assay showed increased NF-κB-Luc activity in Phb1-knockdown RAW 264.7 cells (*p*<0.1). However, SAMe treatments showed no statistical attenuation of NF-κB luciferase activity in LPS stimulated Phb1-knockdown RAW 264.7 cells.

## Discussion

The function of the immune system is to protect the host from infection [16]. These immune systems are divided into innate and adaptive immune system. The innate immune system provides the early line of a defense barrier against infections caused by a variety of stimuli such as LPS and the products of injured cells [17].

Prohibitin 1 is a pleiotropic protein that has multiple functions in cells such as regulation of cell cycle and stabilization of mitochondrial proteins [1, 2]. Despite the several reports implicating Phb1 is involved in immune response, the role of Phb1 in innate immunity remains elusive [2, 18]. Therefore, the main purpose of this study was to investigate the potential immune-modulatory function of Phb1 using a murine macrophage cell line and to discover the anti-inflammatory properties of SAMe.

At first, we elucidate the role of Phb1 in the LPS-induced inflammatory responses by using *siPhb1*. In this study, we confirmed the high significance of *siPhb1* transfection efficiency in RAW 264.7 cells. Interestingly, the mRNA expression level of Phb1 was significantly decreased in LPS-treated RAW 264.7 cells (Fig 1A and 1B). Although the mRNA expression level of *Phb1* in the LPS-treated control group decreased by about 40%, the protein expression level of PHB1 was not significantly different between the normal control group and the LPS-treated control group. This is because PHB1 protein appears to be quite stable, with a half-life that exceeds 10 hours [19], which results in delayed decrease of the PHB1 protein compared to that of the mRNA. Several studies have suggested various role of PHB1 protein in immune functions, such as an association with the IgM receptor in murine B cells or PHB1 protein as a host target protein for pathogens [3]. For that reason, we can hypothesize the impairment of PHB1 function may affect the inflammatory response.

Previous studies showed that the expression of Phb1 not only decreased the inflammatory status but also increased the inflammatory response after infection [20]. Similarly, our study showed that the expression of Phb1 was downregulated by stimulation with LPS in RAW 264.7 cells (Fig 1). Sánchez-Quiles et al (2012) reported that partial global depletion of Phb1 promoted the production of pro-inflammatory cytokines such as TNF-α and IL-1 [21]. Additionally, Theiss et al (2009) reported that Phb1 inhibits TNF-α induced NF-κB activation in intestinal epithelial cells and prohibitin transgenic mice [20]. Considering these findings, our results indicate that there may be a relationship between the inflammatory response and Phb1 expression especially in the acute immune responding cells such as macrophages.

Lipopolysaccharide triggers the production of inflammatory mediators such as TNF-α, interleukins, and inducible nitric oxide synthase (iNOS) by stimulating toll-like receptor 4 (TLR4), which is expressed on the surface of macrophages [22]. TLR4 activates transcription factors, such as NF-κB and interferon regulatory factor (IRF), and ultimately promotes the expression of inflammatory cytokines such as TNF-α, IL-1, and IL-6 [23–25]. These cytokines regulate the level of the immune response and mediate various biological processes. Thus, we examined the LPS-induced inflammatory mediators after knockdown of Phb1 mRNA. In our study, the mRNA expression levels of pro- and anti-inflammatory cytokines were increased by LPS tretments (Fig 3). Intriguingly, the LPS-induced increase in the mRNA expression of TNF-α, IL-6, IL-1α, and IL-1β was significantly further enhanced in the presence of *siPhb1*, while that of IL-10 was significantly attenuated (Fig 2). In the immune system, cytokines are secreted as a response to inflammation in different immune cells. They modulate the inflammatory responses and play key roles in cell survival, growth, and proliferation [26, 27]. TNF-α, IL-1, and IL-6 are known as pro-inflammatory cytokines mostly secreted during the acute phase of inflammation [28]. However, excessive production of pro-inflammatory cytokines is deleterious to the host [29]. For this reason, anti-inflammatory cytokines such as IL-10 and IL-4 inhibit the pro-inflammatory cytokine [30]. Especially, IL-10 is the most important anti-inflammatory cytokine, suppressing the production of LPS-induced pro-inflammatory cytokines in macrophages [31]. Our findings indicate that reduced Phb1 expression can contribute to increased expression of pro-inflammatory cytokines and expansion of the inflammatory response in LPS-induced RAW 264.7 cells. Nos2 gene produces an inducible nitric oxide synthase (iNOS) which is a key enzyme in the synthesis of nitric oxide (NO), an inflammatory mediator that protects the host by removing pathogens [32]. Our results showed that NO production and *Nos2* mRNA expression were significantly increased by LPS stimulation. In addition, LPS-induced NO production and *Nos2* mRNA expression were further enhanced significantly in the presence of *siPhb1*. These results suggest that Phb1 expression level is involved in the LPS-induced inflammatory response.

S-adenosylmethionine (SAMe) is the principal biological methyl donor and has anti-inflammatory properties. In the previous study, we reported SAMe as a potent substance in the regulation of the LPS-induced immune response [32]. Therefore, we next investigated to identify the function of SAMe on LPS-induced inflammatory response related to Phb1. To our knowledge, we first confirmed the effect of SAMe on *Phb1* mRNA expression in LPS-stimulated RAW 264.7 cells. There was no significant difference in the Phb1 expression level after SAMe treatment (Fig 4). Li et al reported that SAMe inhibited LPS-induced TNF-α and IL-1 expression in mice [33]. Another report also showed that SAMe attenuated LPS-induced TNF-α and Nos2 expression and increased IL-10 in a dose-dependent manner. Our results presented that SAMe attenuated LPS-induced IL-1β, IL-10, and Nos2 expression as well as NO production (Figs 5 and 6). Interestingly, SAMe treatment had no effect on LPS-induced TNF-α and IL-6 expression in Phb1 knockdown RAW 264.7 cells (Fig 5A and 5B). In contrast, the mRNA expression levels of IL-1β and IL-10 were reduced by SAMe treatment in LPS-stimulated Phb1 knockdown cells (Fig 5C and 5D). Furthermore, we identified that extracellular secreted TNF-α was significantly decreased in LPS stimulated SAMe treated Phb1 KD group compared to Phb1 expression groups (Fig 5E). These results demonstrated that SAMe has a inmmuno-modulatory effects not on transcriptional level, it has effects on post transcriptional functional property. Based on these results, we concluded that Phb1 modulates the LPS-induced inflammatory response and that SAMe has an anti-inflammatory effect in Phb1 knockdown macrophage cells.

S-adenosylmethionine has been known as an anti-inflammatory compound through NF-κB activation. Theiss et al (2009) reported that Phb1 inhibits TNF-α induced NF-κB activation [20]. Similarly, our results also showed that NF-κB activation is increased by LPS and the activation of NF-κB was more increased in Phb1 KD group. However, SAMe treatments showed no anti-inflammatory effect in LPS stimulated RAW 264.7 cells by measuring NF-κB luciferase activity. These results suggest that without Phb1, NF-κB signaling pathway and its downstream signaling is upregulated and these effects were more sever in LPS stimulated status. Moreover, without NF-κB modulatory effects, SAMe downregulated the mRNA expression level of IL-1β and IL-10 which are downstream signaling of NF-κB. It is well known that NF-κB is present in cells in an inactive state and do not require new protein synthesis in order to become activated. This allows NF-κB to be active by several inducers such as reactive oxygen species, TNF-α, and LPS as a first responder. These results represent that the immune-modulatory effects of SAMe has effects on downstream pathway of NF-κB which would be independent of NF-κB expression changes. In addition we may assume there is a possibility that SAMe regulates phosphorylation or ubiquitination of NF-κB which results in translocation of NF-κB from cytosol to nucleus and modulates transcription level of signaling molecules. However, the mechanisms related to the modulation of the immune response remain unclear.

Taken together, our results show that the level of Phb1 may affect the inflammatory response induced by LPS in murine macrophages. LPS treatment reduces Phb1 mRNA and protein expression in RAW 264.7 cells. Phb1 knockdown further ameliorates the mRNA expression of pro- and anti- inflammatory cytokines such as TNF-α, IL-1α, IL-1β, IL-6, and IL-10 in LPS-stimulated RAW 264.7 cells. In addition to inflammatory cytokines, *Nos2* mRNA expression and NO production are also increased by LPS-stimulation. SAMe treatment attenuates the mRNA expression of LPS-induced inflammatory genes such as IL-1β, IL-10, and Nos2 as well as NO production in the presence of *siPhb1*. LPS stimulation increases NF-κB luciferase activity, which is further increased by Phb1 knockdown. However, SAMe treatment reverts this increase and the mechanism studies will be needed for understanding the specific target of its inflammatory regulation effects. These results demonstrate that Phb1 expression level potentially affects the innate immune system by modulating the NF-κB pathway in RAW 264.7 cells.

## Supporting information

S1 Raw images (PDF)

## References

[1] Merkwirth C, Langer T. Prohibitin function within mitochondria: essential roles for cell proliferation and cristae morphogenesis. Biochimica et biophysica acta. 2009;1793(1):27–32. 10.1016/j.bbamcr.2008.05.013 18558096

[2] Nijtmans LG, Artal SM, Grivell LA, Coates PJ. The mitochondrial PHB complex: roles in mitochondrial respiratory complex assembly, ageing and degenerative disease. Cellular and molecular life sciences: CMLS. 2002;59(1):143–55. 10.1007/s00018-002-8411-0 11852914 PMC11337490

[3] Terashima M, Kim KM, Adachi T, Nielsen PJ, Reth M, Kohler G, et al The IgM antigen receptor of B lymphocytes is associated with prohibitin and a prohibitin-related protein. The EMBO journal. 1994;13(16):3782–92. 8070406 10.1002/j.1460-2075.1994.tb06689.x PMC395291

[4] Lucas CR, Cordero-Nieves HM, Erbe RS, McAlees JW, Bhatia S, Hodes RJ, et al Prohibitins and the cytoplasmic domain of CD86 cooperate to mediate CD86 signaling in B lymphocytes. Journal of immunology (Baltimore, Md: 1950). 2013;190(2):723–36. 10.4049/jimmunol.1201646 23241883 PMC3538926

[5] Kim DK, Kim HS, Kim AR, Jang GH, Kim HW, Park YH, et al The scaffold protein prohibitin is required for antigen-stimulated signaling in mast cells. Science signaling. 2013;6(292):ra80 10.1126/scisignal.2004098 24023254

[6] Ross JA, Nagy ZS, Kirken RA. The PHB1/2 phosphocomplex is required for mitochondrial homeostasis and survival of human T cells. The Journal of biological chemistry. 2008;283(8):4699–713. 10.1074/jbc.M708232200 18086671

[7] Theiss AL, Idell RD, Srinivasan S, Klapproth JM, Jones DP, Merlin D, et al Prohibitin protects against oxidative stress in intestinal epithelial cells. FASEB journal: official publication of the Federation of American Societies for Experimental Biology. 2007;21(1):197–206. 10.1096/fj.06-6801com 17135366

[8] Hsieh SY, Shih TC, Yeh CY, Lin CJ, Chou YY, Lee YS. Comparative proteomic studies on the pathogenesis of human ulcerative colitis. Proteomics. 2006;6(19):5322–31. 10.1002/pmic.200500541 16947118

[9] Theiss AL, Jenkins AK, Okoro NI, Klapproth JM, Merlin D, Sitaraman SV. Prohibitin inhibits tumor necrosis factor alpha-induced nuclear factor-kappa B nuclear translocation via the novel mechanism of decreasing importin alpha3 expression. Molecular biology of the cell. 2009;20(20):4412–23. 10.1091/mbc.e09-05-0361 19710421 PMC2762146

[10] Lu SC. S-Adenosylmethionine. The international journal of biochemistry & cell biology. 2000;32(4):391–5. 10762064 10.1016/s1357-2725(99)00139-9

[11] Pfalzer AC, Choi SW, Tammen SA, Park LK, Bottiglieri T, Parnell LD, et al S-adenosylmethionine mediates inhibition of inflammatory response and changes in DNA methylation in human macrophages. Physiological genomics. 2014;46(17):617–23. 10.1152/physiolgenomics.00056.2014 25180283

[12] Mato JM, Corrales FJ, Lu SC, Avila MA. S-Adenosylmethionine: a control switch that regulates liver function. FASEB journal: official publication of the Federation of American Societies for Experimental Biology. 2002;16(1):15–26. 10.1096/fj.01-0401rev 11772932

[13] Najm WI, Reinsch S, Hoehler F, Tobis JS, Harvey PW. S-adenosyl methionine (SAMe) versus celecoxib for the treatment of osteoarthritis symptoms: a double-blind cross-over trial. [ISRCTN36233495]. BMC musculoskeletal disorders. 2004;5:6 10.1186/1471-2474-5-6 15102339 PMC387830

[14] Gobejishvili L, Avila DV, Barker DF, Ghare S, Henderson D, Brock GN, et al S-adenosylmethionine decreases lipopolysaccharide-induced phosphodiesterase 4B2 and attenuates tumor necrosis factor expression via cAMP/protein kinase A pathway. The Journal of pharmacology and experimental therapeutics. 2011;337(2):433–43. 10.1124/jpet.110.174268 21266552 PMC3083110

[15] Moon MK, Kim M, Chung SS, Lee HJ, Koh SH, Svovoda P, et al S-Adenosyl-L-methionine ameliorates TNFalpha-induced insulin resistance in 3T3-L1 adipocytes. Experimental & molecular medicine. 2010;42(5):345–52. 10.3858/emm.2010.42.5.036 20208423 PMC2877253

[16] Parkin J, Cohen B. An overview of the immune system. Lancet (London, England). 2001;357(9270):1777–89. 10.1016/S0140-6736(00)04904-7 11403834

[17] Beutler B. Innate immunity: an overview. Molecular immunology. 2004;40(12):845–59. 10.1016/j.molimm.2003.10.005 14698223

[18] Theiss AL, Laroui H, Obertone TS, Chowdhury I, Thompson WE, Merlin D, et al Nanoparticle-based therapeutic delivery of prohibitin to the colonic epithelial cells ameliorates acute murine colitis. Inflammatory bowel diseases. 2011;17(5):1163–76. 10.1002/ibd.21469 20872832 PMC3012155

[19] He B, Feng Q, Mukherjee A, Lonard DM, DeMayo FJ, Katzenellenbogen BS, et al A repressive role for prohibitin in estrogen signaling. Molecular endocrinology (Baltimore, Md). 2008;22(2):344–60. 10.1210/me.2007-0400 17932104 PMC2234581

[20] Sanchez-Quiles V, Segura V, Bigaud E, He B, O’Malley BW, Santamaria E, et al Prohibitin-1 deficiency promotes inflammation and increases sensitivity to liver injury. Journal of proteomics. 2012;75(18):5783–92. 10.1016/j.jprot.2012.08.009 22951295

[21] Hoebe K, Janssen E, Beutler B. The interface between innate and adaptive immunity. Nature immunology. 2004;5(10):971 10.1038/ni1004-971 15454919

[22] Medzhitov R. Toll-like receptors and innate immunity. Nature reviews Immunology. 2001;1(2):135–45. 10.1038/35100529 11905821

[23] Raetz CR, Whitfield C. Lipopolysaccharide endotoxins. Annual review of biochemistry. 2002;71:635–700. 10.1146/annurev.biochem.71.110601.135414 12045108 PMC2569852

[24] Lu YC, Yeh WC, Ohashi PS. LPS/TLR4 signal transduction pathway. Cytokine. 2008;42(2):145–51. 10.1016/j.cyto.2008.01.006 18304834

[25] Zhang J-M, An J. Cytokines, inflammation and pain. International anesthesiology clinics. 2007;45(2):27 10.1097/AIA.0b013e318034194e 17426506 PMC2785020

[26] Luster AD. Chemokines—chemotactic cytokines that mediate inflammation. The New England journal of medicine. 1998;338(7):436–45. 10.1056/NEJM199802123380706 9459648

[27] Scheller J, Chalaris A, Schmidt-Arras D, Rose-John S. The pro- and anti-inflammatory properties of the cytokine interleukin-6. Biochimica et biophysica acta. 2011;1813(5):878–88. 10.1016/j.bbamcr.2011.01.034 21296109

[28] Dinarello CA. Proinflammatory cytokines. Chest. 2000;118(2):503–8. 10.1378/chest.118.2.503 10936147

[29] Iyer SS, Cheng G. Role of interleukin 10 transcriptional regulation in inflammation and autoimmune disease. Critical reviews in immunology. 2012;32(1):23–63. 10.1615/critrevimmunol.v32.i1.30 22428854 PMC3410706

[30] Fiorentino DF, Zlotnik A, Mosmann TR, Howard M, O’Garra A. IL-10 inhibits cytokine production by activated macrophages. Journal of immunology (Baltimore, Md: 1950). 1991;147(11):3815–22. 1940369

[31] Alderton WK, Cooper CE, Knowles RG. Nitric oxide synthases: structure, function and inhibition. The Biochemical journal. 2001;357(Pt 3):593–615. 10.1042/0264-6021:3570593 11463332 PMC1221991

[32] Lee SY, Ko KS. Protective Effects of S-Adenosylmethionine and Its Combinations With Taurine and/or Betaine Against Lipopolysaccharide or Polyinosinic-polycytidylic Acid-induced Acute Hepatotoxicity. Journal of cancer prevention. 2016;21(3):152–63. 10.15430/JCP.2016.21.3.152 27722141 PMC5051589

[33] Li P, Zhang Z, Gong J, Zhang Y, Zhu X. S-Adenosylmethionine attenuates lipopolysaccharide induced liver injury by downregulating the Toll-like receptor 4 signaling in Kupffer cells. Hepatology International. 2014;8:275–84 26202509 10.1007/s12072-014-9528-6

