# Supplementary figures and images for: Lipopolysaccharide-induced innate immune responses are exacerbated by Prohibitin 1 deficiency and mitigated by S-adenosylmethionine in murine macrophages
Source: PLoS One. 2020 Nov 11;15(11):e0241224. doi: 10.1371/journal.pone.0241224 (PMC7657527; doi:10.1371/journal.pone.0241224)

PHB1

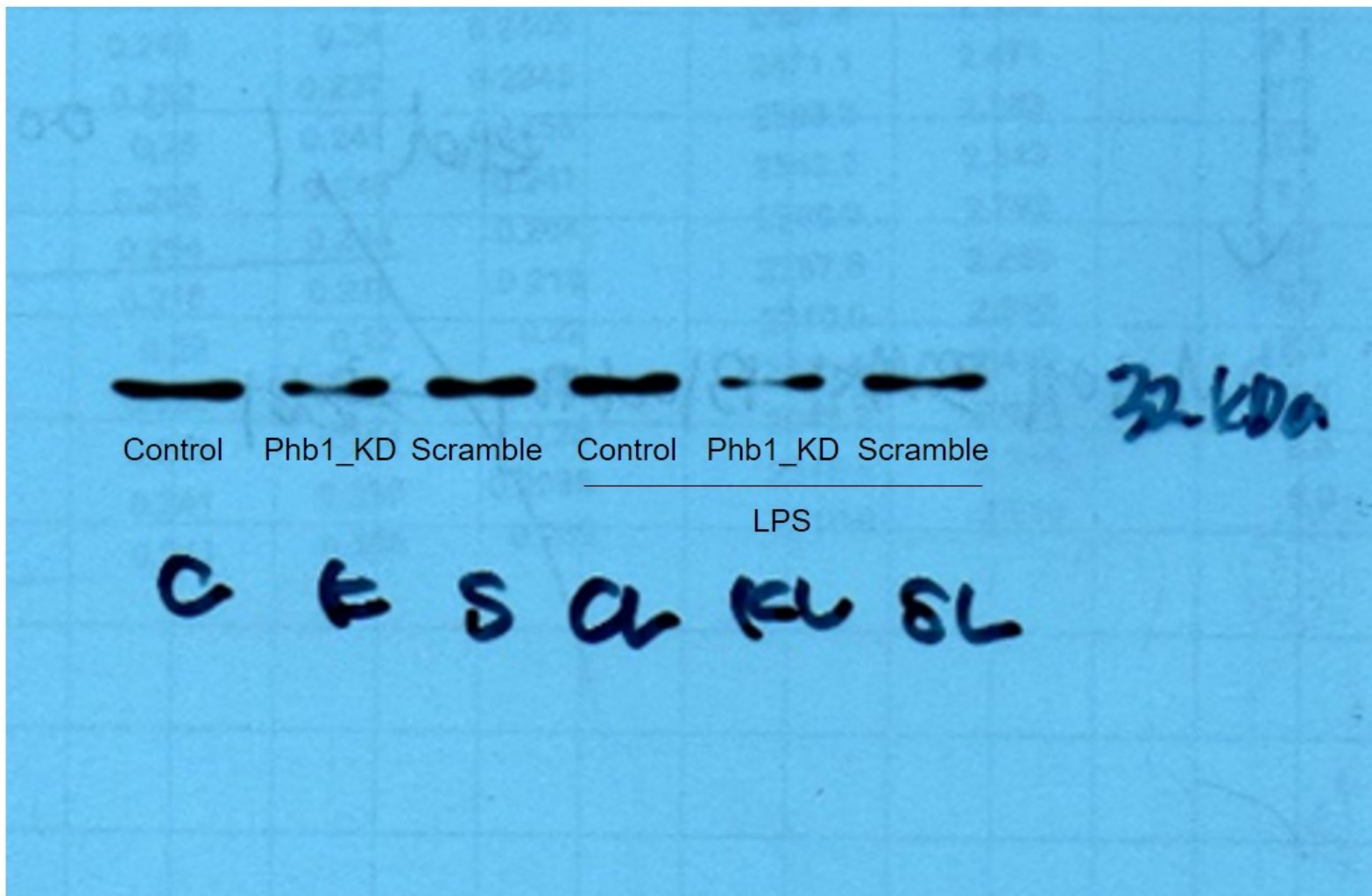

Alpha, tubulin

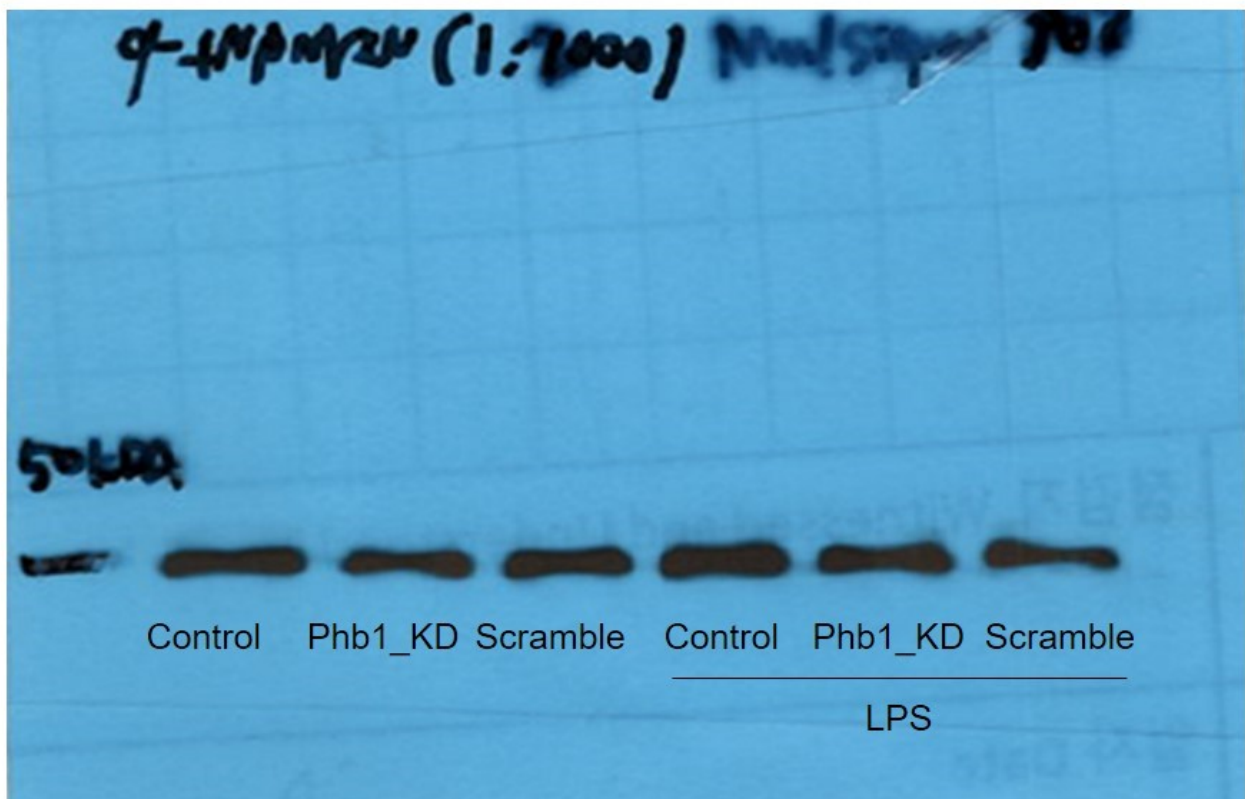

Supplement: S1 Raw images — (PDF) [file pone.0241224.s001.pdf]
